# Supplementary material for: Chronic cough relief by allosteric modulation of P2X3 without taste disturbance
Source: Nat Commun. 2023 Sep 20;14:5844. doi: 10.1038/s41467-023-41495-0 (PMC10511716; doi:10.1038/s41467-023-41495-0)
Supplement: Supplementary file 1 — Supplementary Information [file 41467_2023_41495_MOESM1_ESM.pdf]

Chronic cough relief by allosteric modulation of P2X3 without taste disturbance

Chang-Run Guo[1], Zhong-Zhe Zhang[1], Xing Zhou[1], Meng-Yang Sun[1], Tian-Tian Li[1], Yun-Tao Lei[1], Yu-Hao Gao[1], Qing-Quan Li[1], Chen-Xi Yue[1], Yu Gao[1], Yi-Yu Lin[1], Cui-Yun Hao[1], Chang-Zhu Li[2], Peng Cao[3], Michael X. Zhu[4], Ming-Qiang Rong[5, *], Wen-Hui Wang[1, *], Ye Yu[1, *]

[1]Schools of Basic Medicine and Clinical Pharmacy and Traditional Chinese Pharmacy, and State Key Laboratory of Natural Medicines, China Pharmaceutical University, Nanjing, 211198, China; [2]State Key Laboratory of Utilization of Woody Oil Resource, Hunan Academy of Forestry, Changsha, Hunan 410004; [3]Hospital of Integrated Traditional Chinese and Western Medicine, Nanjing University of Chinese Medicine, Nanjing 210023; [4]Department of Integrative Biology and Pharmacology, McGovern Medical School, The University of Texas Health Science Center at Houston, Houston, Texas 77030. [5]The National & Local Joint Engineering Laboratory of Animal Peptide Drug Development, College of Life Sciences, Hunan Normal University, Changsha, China.

These authors contributed equally: Chang-Run Guo, Zhong-Zhe Zhang, Xing Zhou

[*]correspondence and requests for materials should be addressed to M.Q.R. (rongmq@hunnu.edu.cn), W.H.W. (whwang@cpu.edu.cn) or Y.Y. (yuye@cpu.edu.cn)

This PDF file includes:

Supplementary Figures 1-8

Supplementary Tables 1-2

Supplementary References 1-4

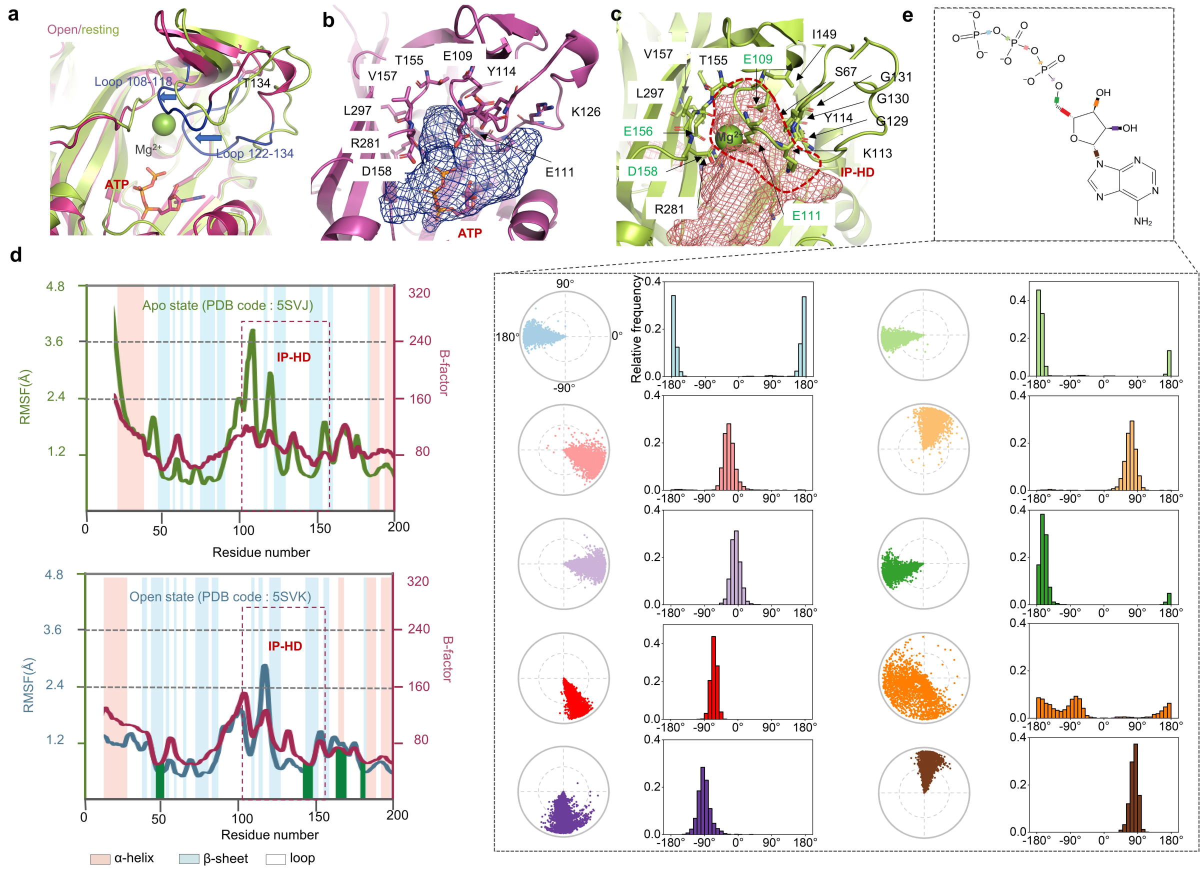

Supplementary Figure 1. The loops 108 - 118 and 122 - 134 in hP2X3 lower head domain are tied up due to the presence of ATP. (**a**) The volume of the internal pocket of the head domain (IP-HD) underwent a significant decrease due to the forced leftward shift of loop 108-118 and loop 122-134. (**b,c**) Zoomed-in view of the cavity formed by the left flipper (LF), dorsal fin (DF) and head domains in the resting (b) and open (c) states. Residues that make up IP-HD are indicated by sticks for emphasis. The green font represents the residues corresponding to the receptor that interacts with the Mg^{2+}. (**d**) Molecular dynamics (MD) simulations of the resting (upper panel) and open (lower panel) hP2X3 structures for about 300-ns showed that the r.m.s. fluctuations (RMSF) of these two loops in the open state were smaller than those of the resting structure. (**e**) The rotation of the individual chemical bonds of ATP was limited to a certain degree of fluctuations throughout simulations. Two-dimensional schematics of ATP are shown as color-coded rotatable bonds. The radial plots represent the conformation of the torsion bodies. The center of the radial plot represents the beginning of the simulation, plotting the temporal evolution in the radial direction outward. The histogram summarizes the data of the corresponding radial plot, which represents the probability density of the torsion.

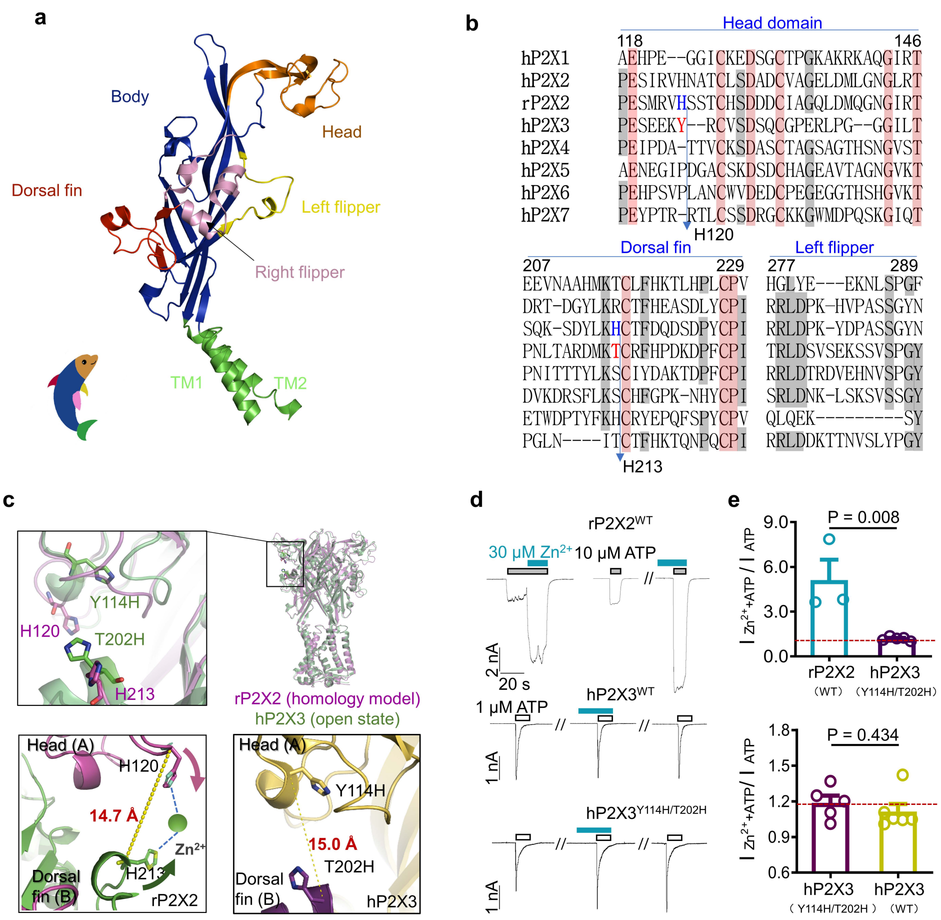

Supplementary Figure 2. Different conformational changes of IP-HD during the activation of P2X2 and P2X3. (**a**) Cartoon representation of the 'dolphin-like' single subunit of the hP2X3 structure. (**b**) The amino acid sequence alignment of residues in the head, dorsal fin (DF) and left flipper (LF) domains of various P2X subtypes. (**c**) The superimposed structures of rP2X2 and hP2X3$^{Y114H/T202H}$ (upper panel). Zoomed-in view of the ATP binding site jaw in rP2X2 (lower left panel) and hP2X3$^{Y114H/T202H}$ (lower right panel). Yellow and green dashed lines represent the measured C_α-C_α distance and Zn^{2+} bridge formed between histidine and Zn^{2+}, respectively. (**d, e**) Representative current traces and pooled data on the ATP-induced activation of (wild type) WT rP2X2, WT hP2X3 and hP2X3$^{Y114H/T202H}$. Each circle represents an individual cell, n = 3 (rP2X2), 5 (hP2X3$^{Y114H/T202H}$) or 6 (hP2X3^{WT}); unpaired two-tailed t-test. All summary data are expressed as mean ± SEM. Source data are provided as a Source Data file.

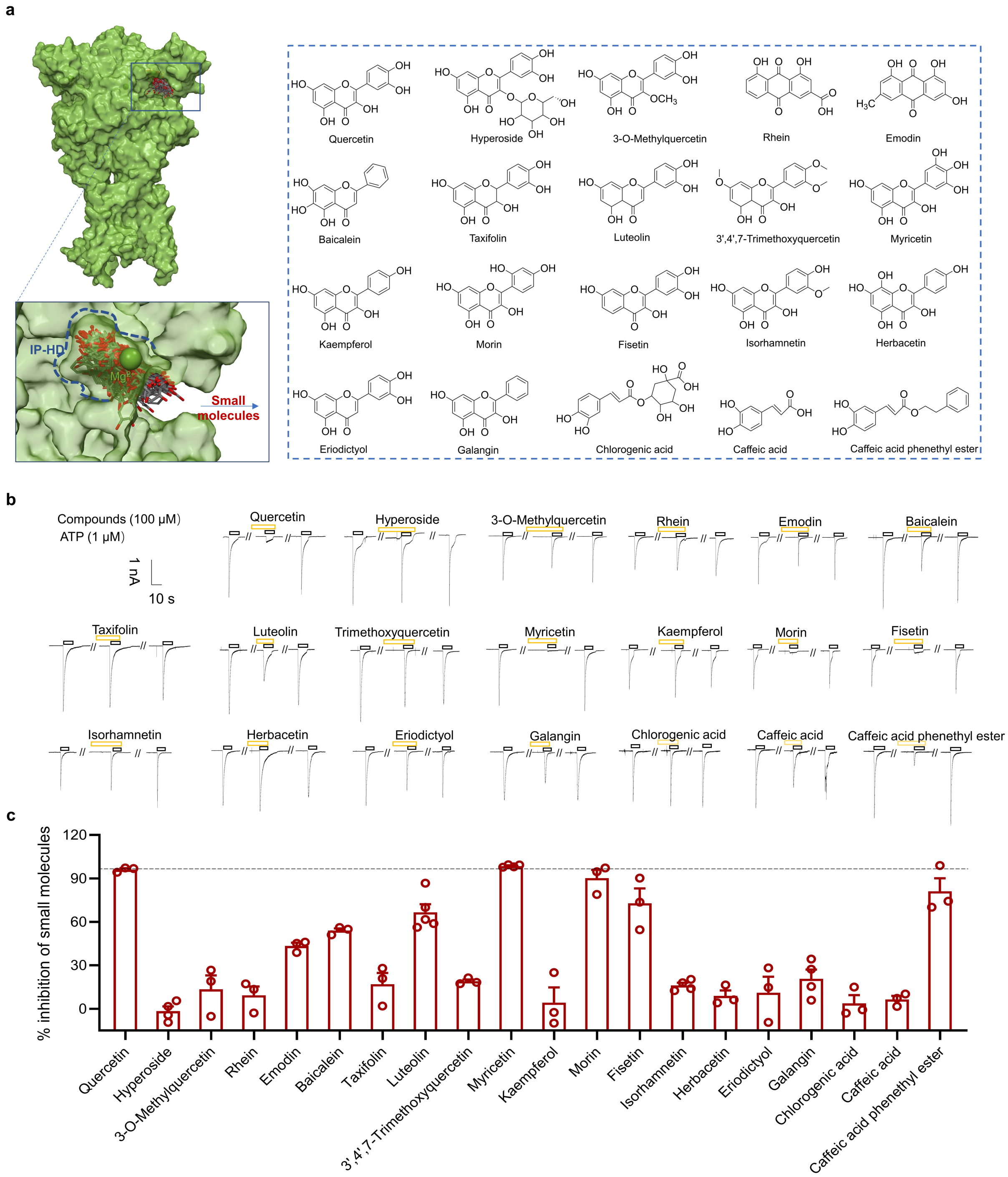

Supplementary Figure 3. Screening the P2X3 inhibitors based on the IP-HD tightening. (**a**) Chemical structures of screened compounds. (**b, c**) Typical current traces (b) and pooled data (c) showing the effect of chemicals (100 µM) on hP2X3 receptors. Each circle represents an independent cell; n = 3 (quercetin, 3-O-methylquercetin, rhein, emodin, baicalein, taxifolin, 3',4',7-trimethoxyquercetin, kaempferol, morin, fisetin, herbacetin, eriodictyol, chlorogenic acid, caffeic acid, and caffeic acid phenethyl ester), 4 (hyperoside, myricetin, galangin, and isorhamnetin) or 5 (luteolin) All summary data are expressed as mean ± SEM. Source data are provided as a Source Data file.

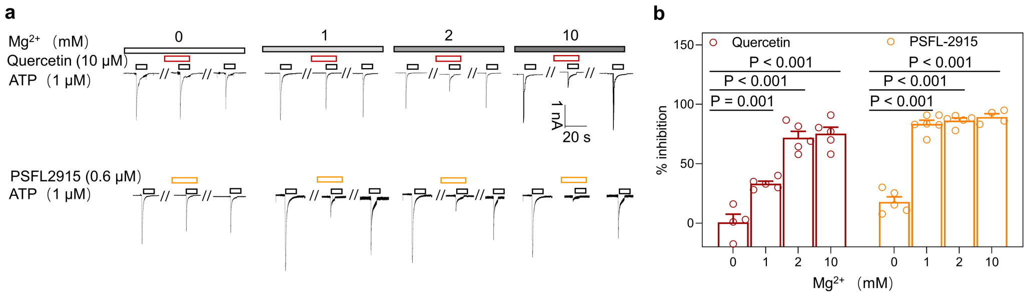

Supplementary Figure 4. Extracellular Mg^{2+} concentration contributes to the inhibition of quercetin and PSFL2915 on P2X3 receptors. (a, b) Typical current traces (a) and pooled data (b) showing the mechanism of inhibition of quercetin and PSFL2915 by Mg^{2+}. Each circle represents an individual cell, n = 4 (0 mM Mg^{2+}) or 5 (1, 2, and 10 mM Mg^{2+}) for quercetin; n = 4 (10 mM Mg^{2+}), 5 (0 and 2 mM Mg^{2+}) or 6 (1 mM Mg^{2+}) for PSFL2915; one-way ANOVA followed by Dunnett's multiple comparisons test, F (3,15) = 45.46. and F (3,16) = 111.3, respectively. All summary data are expressed as mean ± SEM. Source data are provided as a Source Data file.

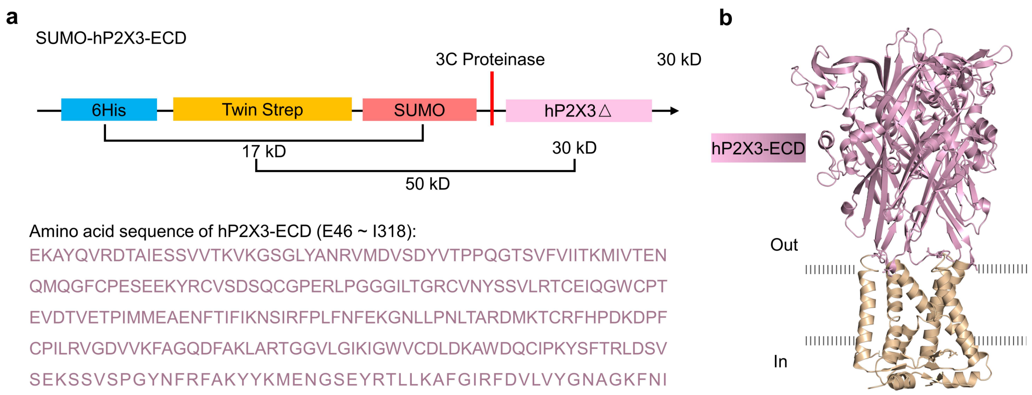

Supplementary Figure 5. Strategies used to purify the extracellular domain of hP2X3 receptors (hP2X3-ECD). (**a**) Design of the hP2X3-ECD expression system (top panel) and its amino acid sequence (bottom panel). (**b**) Side view of hP2X3-ECD.

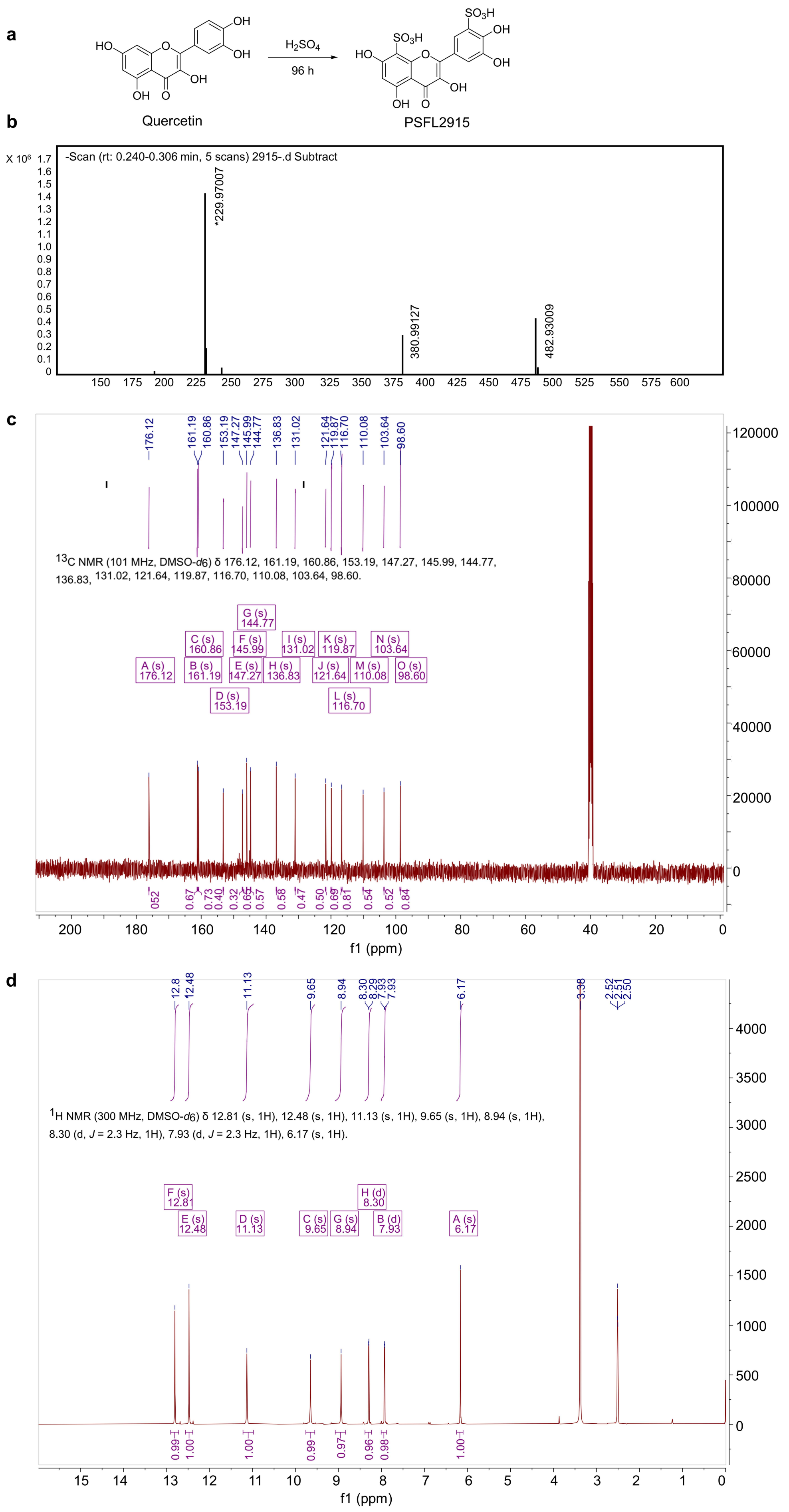

Supplementary Figure 6. Chemical synthesis of PSFL2915. (a-d) Chemical synthesis of PSFL2915 (a), and its high-resolution mass spectral (b), ^{13}C NMR (100 MHz, DMSO - *d*6) (c), and ^{1}H NMR (300 MHz, DMSO - *d*6) spectra (d).

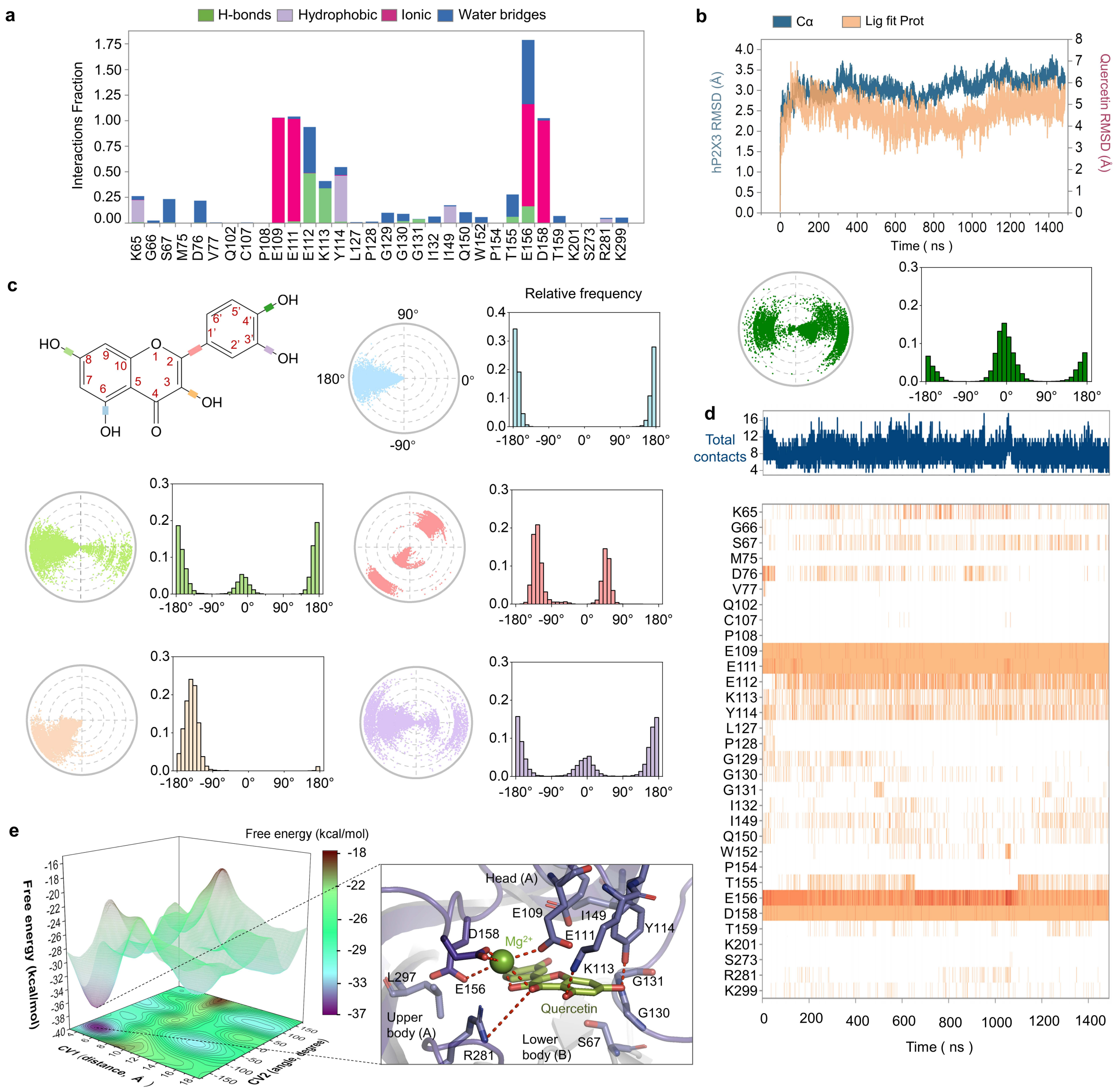

Supplementary Figure 7. Conventional (molecular dynamics, MD) and enhanced samplings (metadynamics, MetaD) simulations to study the interaction of hP2X3 with quercetin at the atomic level. (**a**) The interaction between P2X3 and quercetin was monitored throughout the MD simulations. hP2X3/quercetin interactions were classified into four types: hydrogen bonds (green), hydrophobic (light purple), Mg^{2+}-assisted ionic interactions, pink), and water bridges (blue). The stacked histograms are normalized over the course of the trajectory. (**b**) Backbone root-mean-square deviation (RMSD) analysis of the binding process of quercetin to hP2X3 throughout the MD simulation (0-1500 ns). (**c**) Torsion plots of quercetin summarizing the conformational evolution of each rotatable bond every 10 ns throughout the simulated trajectory. Two-dimensional schematics of quercetin are shown as color-coded rotatable bonds. The radial plots represent the conformation of the torsion bodies. The center of the radial plot represents the beginning of the simulation, plotting the temporal evolution in the radial direction outward. The histogram summarizes the data of the corresponding radial plot, which represents the probability density of the torsion. The relationship between histogram and torsional potential gives insight into the conformational strain that the ligand underwent to maintain the hP2X3-bound conformation. (**d**)Time-dependent contacts between P2X3 and quercetin throughout the MD simulations. The upper panel represents the total contacts of P2X3 with quercetin every 200 ps throughout the simulation. The lower panel represents the contacts of single amino acid with quercetin. The darker orange color indicates the number of residues interacting with the ligand, as some amino acids have multiple specific contracts with the ligand. (**e**) Three-dimensional free-energy reconstruction of the interaction between quercetin and hP2X3 (left), and the best conformation of quercetin bound to hP2X3 based on MetaD-enhanced conformational sampling (right). Red dashed lines represent important polar interactions, including hydrogen bonds and Mg^{2+}-assisted ionic interactions.

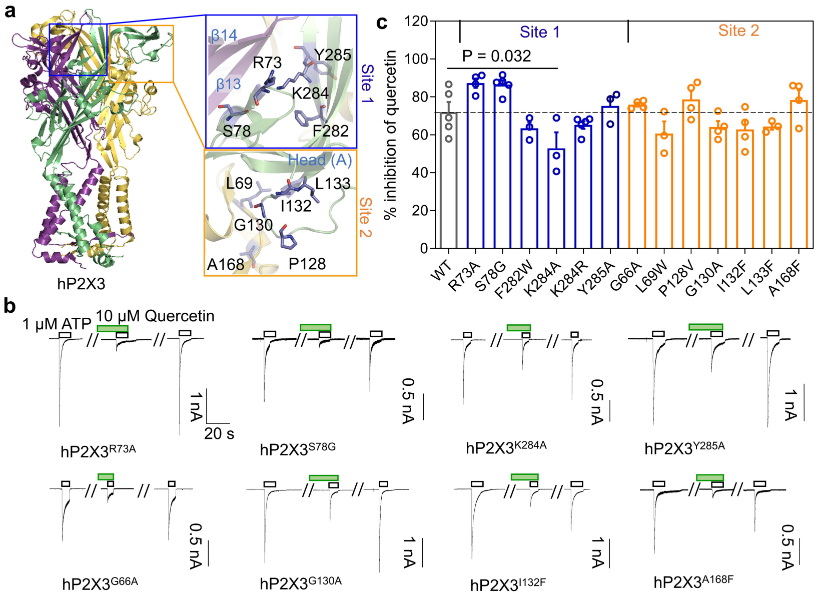

Supplementary Figure 8. Other possible sites for quercetin in the P2X3 receptor. (**a**) Zoomed-in view of the possible interaction sites 1 and 2 of quercetin in hP2X3 Residues forming these pockets are indicated by sticks for emphasis. (**b**, **c**) Representative current traces (b) and pooled data (c) showing the effect of quercetin (10 μM) on ATP (1 μM)-induced cellular activation, indicating WT hP2X3 and its mutants. Each circle represents an independent cell. n = 3 (L69W, L133F, F282W, K284A, and Y285A), 4 (G66A, R73A, P128V, G130A, I132F, and A168F) or 5 (WT, S78G, and K284R); One-way ANOVA followed by Dunnett's multiple comparison test, $F(13, 40) = 5.711$. Data are expressed as mean ±SEM. Source data are provided as a Source Data file.

Supplemental Table 1. The structure and perhaps in vitro pharmacological properties of inhibitors targeting P2X3 receptors.

Compounds	Structure	hP2X3 IC_{50}
S-600918 [a]	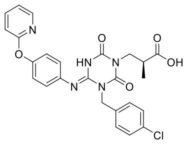	67.5 ± 1.2 nM [1]
Gefapixant [b]	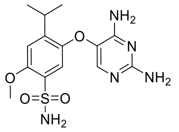	76 nM [2]
BLU-5937 [b]	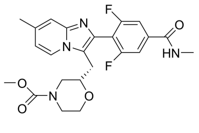	25 nM [3]
Eliapixant [c]	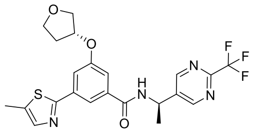	8 nM [4]

Agonists: [a] 1 μM ATP; [b] 3 μM α-β-methylene ATP; [c] 10 μM α-β-methylene ATP.

Supplementary Table 2. Primers used in this study to generate point mutations

Name	Primer-Forward	Primer-Reverse
hP2X3-V143ANAP	TTACCTGGTGGGTAGATCCTCACTGGCCGC	CTCACAGGTCCGGAGCTAAGAACTGTAGTT
hP2X3-E111ANAP	GGGGGGATCCTCTAGGGCCGCTGCGTGAAC	ACAGCGGTATTTCTCCTAACTCTCTGGGCA
hP2X3-Y114ANAP	CGGACCTGTGAGTAGCAGGGCTGGTGCCCC	GTCTGATACACAGCGCTATTTCTCCTCACT
hP2X3-G131ANAP	TGGTGCCCCACGTAGGTGGACACAGTGGAA	GCGGCCAGTGAGGATCTACCCACCAGGTAA
hP2X3-T134ANAP	GGCTACAACTTCTAGTTTGCCAAGTACTAC	GTTCACGCAGCGGCCCTAGAGGATCCCCCC
hP2X3-I149ANAP	GAGTACCGCACCTAGCTGAAGGCTTTTGGC	GGGGCACCAGCCCTGCTACTCACAGGTCCG
hP2X3-E156ANAP	CCCACGGAGGTGTGTACAGTGGAAACGCCC	TTCCACTGTGTCCACCTACGTGGGGCACCA
hP2X3-R281ANAP	TTACCTGGTGGGTAGATCCTCACTGGCCGC	GTAGTACTTGGCAAACTAGAAGTTGTAGCC
hP2X3-L297ANAP	GGGGGGATCCTCTAGGGCCGCTGCGTGAAC	GCCAAAAGCCTTCAGCTAGGTGCGGTACTC
hP2X3-D158C	CGGACCTGTGAGTAGCAGGGCTGGTGCCCC	GGGCGTTTCCACTGTACACACCTCCGTGGG
hP2X3-E111C	TGCCCAGAGAGTTGTGAGAAATACCGCTGT	ACAGCGGTATTTCTCACAACTCTCTGGGCA
hP2X3-L127C	GGGCCTGAGCGCTGTCCAGGTGGGGGGATC	GATCCCCCCACCTGGACAGCGCTCAGGCCC
hP2X3-T202C	AGGGACATGAAGTGTTGCCGCTTCCACCCG	CGGGTGGAAGCGGCAACACTTCATGTCCCT
hP2X3-G131C	TTGCCAGGTGGGTGTATCCTCACTGGCCGC	GCGGCCAGTGAGGATACACCCACCTGGCAA
hP2X3-L127H	GGGCCTGAGCGCCATCCAGGTGGGGGGATC	GATCCCCCCACCTGGATGGCGCTCAGGCCC
hP2X3-T202H	AGGGACATGAAACACTGCCGCTTCCACCCG	CGGGTGGAAGCGGCAGTGTTTCATGTCCCT
hP2X3-V77C	AGAGTCATGGATTGTTCTGATTACGTGACG	CGTCACGTAATCAGAACAATCCATGACTCT
hP2X3-D158C	TGCCCCACGGAGGTGTGTACAGTGGAAACG	CGTTTCCACTGTACACACCTCCGTGGGGCA
hP2X3-I149C	CTCCGGACCTGTGAGTGTCAGGGCTGGTGC	GCACCAGCCCTGACACTCACAGGTCCGGAG
hP2X3-E156C	TGGTGCCCCACGTGTGTGGACACAGTGGAA	TTCCACTGTGTCCACACACGTGGGGCACCA
hP2X3-S67F	AAGGTGAAGGGCTTCGGACTCTACGCAAAC	GTTTGCGTAGAGTCCGAAGCCCTTCACCTT
hP2X3-S67W	AAGGTGAAGGGCTGGGGACTCTACGCAAAC	GTTTGCGTAGAGTCCCCAGCCCTTCACCTT
hP2X3-E109A	GGATTCTGCCCAGCAAGTGAGGAGAAATAC	GTATTTCTCCTCACTTGCTGGGCAGAATCC

hP2X3-S110F	TTCTGCCCAGAGTTTGAGGAGAAATACCGC	GCGGTATTTCTCCTCAAACTCTGGGCAGAA
hP2X3-E111I	TGCCCAGAGAGTATAGAGAAATACCGCTGT	ACAGCGGTATTTCTCTATACTCTCTGGGCA
hP2X3-K113F	GAGAGTGAGGAGGCATACCGCTGTGTATCA	TGATACACAGCGGTATGCCTCCTCACTCTC
hP2X3-Y114A	AGTGAGGAGAAAGCCCGCTGTGTATCAGAC	GTCTGATACACAGCGGGCTTTCTCCTCACT
hP2X3-G129P	GAGCGCTTGCCACCTGGGGGGATCCTCACT	AGTGAGGATCCCCCCAGGTGGCAAGCGCTC
hP2X3-G131W	TTGCCAGGTGGGTGGATCCTCACTGGCCGC	GCGGCCAGTGAGGATCCACCCACCTGGCAA
hP2X3-I149F	CGGACCTGTGAGTTTCAGGGCTGGTGCCCC	GGGGCACCAGCCCTGAAACTCACAGGTCCG
hP2X3-E156A	TGGTGCCCCACGGCAGTGGACACAGTGGAA	TTCCACTGTGTCCACTGCCGTGGGGCACCA
hP2X3-E156F	TGGTGCCCCACGTTTGTGGACACAGTGGAA	TTCCACTGTGTCTACAAACGTGGGGCACCA
hP2X3-D158A	CCCCACGGAGGTGGCCACAGTAGAAAC	GTTTCTACTGTGGCCACCTCCGTGGGG
hP2X3-L297A	GAGTACCGCACCGCCCTGAAGGCTTTTGGC	GCCAAAAGCCTTCAGGGCGGTGCGGTACTC
hP2X3-L297W	GAGTACCGCACCTGGCTGAAGGCTTTTGGC	GCCAAAAGCCTTCAGCCAGGTGCGGTACTC
hP2X3-L297C	GAGTACCGCACCTGTCTGAAGGCTTTTGGC	GCCAAAAGCCTTCAGACAGGTGCGGTACTC
hP2X3-Y114H	AGTGAGGAGAAACACCGCTGTGTATCAGAC	GTCTGATACACAGCGGTGTTTCTCCTCACT
hP2X3-R73A	CTCTACGCCAACGCAGTCATGGATGTGTCT	AGACACATCCATGACTGCGTTGGCGTAGAG
hP2X3-S78G	AGAGTCATGGATGTGGGTGATTACGTG	CACGTAATCACCCACATCCATGACTCT
hP2X3-F282W	TACAACTTCAGGTGGGCCAAGTACTAC	GTAGTACTTGGCCCACCTGAAGTTGTA
hP2X3-K284A	AACTTCAGGTTTGCCGCGTACTACAAAATG	CATTTTGTAGTACGCGGCAAACCTGAAGTT
hP2X3-K284R	AACTTCAGGTTTGCCAGGTACTACAAAATG	CATTTTGTAGTACCTGGCAAACCTGAAGTT
hP2X3-Y285A	TTCAGGTTTGCCAAGGCCTACAAAATGGAA	TTCCATTTTGTAGGCCTTGGCAAACCTGAA
hP2X3-G66A	GTAACCAAGGTGAAGGCCTCCGGACTCTAC	GTAGAGTCCGGAGGCCTTCACCTTGGTTAC
hP2X3-L69W	AAGGGCTCCGGATGGTACGCAAACAGAGTC	GACTCTGTTTGCGTACCATCCGGAGCCCTT
hP2X3-P128V	CCTGAGCGCTTGGTAGGTGGGGGATCCTC	GAGGATCCCCCACCTACCAAGCGCTCAGG
hP2X3-G130A	GAGCGCTTGCCAGGTGCAGGGATCCTCACT	AGTGAGGATCCCTGCACCTGGCAAGCGCTC

hP2X3-I132F	AGGTGGGGGGTTCCTCACTGGCCGCTGCGT	ACGCAGCGGCCAGTGAGGAACCCCCCACCT
hP2X3-L133F	GGTGGGGGGATCTTCACTGGCCGCTGCGTG	CACGCAGCGGCCAGTGAAGATCCCCCCACC
hP2X3-A168F	ATCATGATGGAATTTGAGAACTTCACTATT	AATAGTGAAGTTCTCAAATTCCATCATGAT
hP2X3-Y37A	CTGATCATCTCCGCATTTGTAGGGTGGGTT	AACCCACCCTACAAATGCGGAGATGATCAG

Supplemental References:

1. Dong-Ping Wang, Meng Zhang, Ming Li, et al. Druggable site near the upper vestibule determines the high affinity and P2X3 selectivity of Sivopixant, a clinical candidate for refractory chronic cough with low taste disturbances. *Authorea*. January 31, 2023. DOI: 10.22541/au.167517203.33744065/v1.
2. McGarvey, L.P. et al. Efficacy and safety of gefapixant, a P2X3 receptor antagonist, in refractory chronic cough and unexplained chronic cough (COUGH-1 and COUGH-2): results from two double-blind, randomised, parallel-group, placebo-controlled, phase 3 trials. *Lancet* **399**, 909-923 (2022).
3. Garceau, D. & Chauret, N. BLU-5937: A selective P2X3 antagonist with potent anti-tussive effect and no taste alteration. *Pulm Pharmacol Ther* **56**, 56-62 (2019).
4. Davenport, A.J. et al. Eliapixant is a selective P2X3 receptor antagonist for the treatment of disorders associated with hypersensitive nerve fibers. *Sci Rep* **11**(2021).
