# Supplementary material for: Chronic cough relief by allosteric modulation of P2X3 without taste disturbance
Source: Nat Commun. 2023 Sep 20;14:5844. doi: 10.1038/s41467-023-41495-0 (PMC10511716; doi:10.1038/s41467-023-41495-0)
Supplement: Supplementary file 3 — Reporting Summary [file 41467_2023_41495_MOESM3_ESM.pdf]

nature portfolio

Corresponding author(s): Ye Yu, Wen-Hui Wang, Ming-Qiang Rong

Last updated by author(s): Aug 17, 2023

Reporting Summary

Nature Portfolio wishes to improve the reproducibility of the work that we publish. This form provides structure for consistency and transparency in reporting. For further information on Nature Portfolio policies, see our Editorial Policies and the Editorial Policy Checklist.

Statistics

For all statistical analyses, confirm that the following items are present in the figure legend, table legend, main text, or Methods section.

n/a	Confirmed	
☐	☒	The exact sample size (*n*) for each experimental group/condition, given as a discrete number and unit of measurement
☐	☒	A statement on whether measurements were taken from distinct samples or whether the same sample was measured repeatedly
☐	☒	The statistical test(s) used AND whether they are one- or two-sided<br>*Only common tests should be described solely by name; describe more complex techniques in the Methods section.*
☒	☐	A description of all covariates tested
☐	☒	A description of any assumptions or corrections, such as tests of normality and adjustment for multiple comparisons
☐	☒	A full description of the statistical parameters including central tendency (e.g. means) or other basic estimates (e.g. regression coefficient) AND variation (e.g. standard deviation) or associated estimates of uncertainty (e.g. confidence intervals)
☐	☒	For null hypothesis testing, the test statistic (e.g. *F*, *t*, *r*) with confidence intervals, effect sizes, degrees of freedom and *P* value noted<br>*Give P values as exact values whenever suitable.*
☒	☐	For Bayesian analysis, information on the choice of priors and Markov chain Monte Carlo settings
☒	☐	For hierarchical and complex designs, identification of the appropriate level for tests and full reporting of outcomes
☒	☐	Estimates of effect sizes (e.g. Cohen's *d*, Pearson's *r*), indicating how they were calculated

Our web collection on statistics for biologists contains articles on many of the points above.

Software and code

Policy information about availability of computer code

Data collection	Electrophysiological recordings were collected using Clampfit 10.6 (Molecular Devices).Microscale Thermophoresis data were collected using Monolith NT.115 instrument (NanoTemper Technologies). ChemiDoc Imaging System (Tanon, 5200) was used for Western blot. MetaMorph (v7.8.0.0) microscopy automation and image analysis software (Molecular Devices) was used for voltage clamp fluorometry.
Data analysis	Electrophysiological recordings were analyzed using Clampfit 10.6. Microscale Thermophoresis data were analyzed by Nanotemper analysis software (v2.2.4). Conventional Molecular Dynamics simulations and metadynamics simulations were constructed using DESMOND System(v11.8). GraphPad Prism version 8 software was used to determine statistical significance. Voltage clamp fluorometry data were analyzed by MetaMorph (v7.8.0.0) microscopy automation and image analysis software (Molecular Devices). Homology model was constructed with MODELLER (v10.4), and the quality of the models was checked with ProCheck (v3.4.3).

For manuscripts utilizing custom algorithms or software that are central to the research but not yet described in published literature, software must be made available to editors and reviewers. We strongly encourage code deposition in a community repository (e.g. GitHub). See the Nature Portfolio guidelines for submitting code & software for further information.

Data

Policy information about availability of data

All manuscripts must include a data availability statement. This statement should provide the following information, where applicable:

- Accession codes, unique identifiers, or web links for publicly available datasets
- A description of any restrictions on data availability
- For clinical datasets or third party data, please ensure that the statement adheres to our policy

All experimental data generated or analyzed during this study are presented in this paper and original data electronic copy is available from the corresponding author on reasonable request.

Research involving human participants, their data, or biological material

Policy information about studies with human participants or human data. See also policy information about sex, gender (identity/presentation), and sexual orientation and race, ethnicity and racism.

Reporting on sex and gender	N/A
Reporting on race, ethnicity, or other socially relevant groupings	N/A
Population characteristics	N/A
Recruitment	N/A
Ethics oversight	N/A

Note that full information on the approval of the study protocol must also be provided in the manuscript.

Field-specific reporting

Please select the one below that is the best fit for your research. If you are not sure, read the appropriate sections before making your selection.

☒ Life sciences ☐ Behavioural & social sciences ☐ Ecological, evolutionary & environmental sciences

For a reference copy of the document with all sections, see nature.com/documents/nr-reporting-summary-flat.pdf

Life sciences study design

All studies must disclose on these points even when the disclosure is negative.

Sample size	No statistical method was used to calculate sample size. Sample sizes were chosen based on standard practices in this field of research and previous analyses and experience with similar experimental paradigms (PMID:36546250, PMID:29674445, PMID: 30902655, PMID: 30721152)
Data exclusions	No data were excluded.
Replication	All in vitro experiments were repeated independently at least three times. All in vivo experiments and its detections were performed using a minimum of 7 animals. All replication attempts were successful.
Randomization	Animals were randomly assigned to experimental groups at the start of the study. For in vitro experiments, cells were equally distributed into culture dishes and followed by different treatments with replications, no randomization was required.
Blinding	The group allocation of animals was conducted in a blinded manner.<br>The experimental results analysis of molecular biology and electrophysiological are obtained by quantitative methods, so we were not blinded to sample allocation.

Reporting for specific materials, systems and methods

We require information from authors about some types of materials, experimental systems and methods used in many studies. Here, indicate whether each material, system or method listed is relevant to your study. If you are not sure if a list item applies to your research, read the appropriate section before selecting a response.

Materials & experimental systems

n/a	Involved in the study	
☐	☒	Antibodies
☐	☒	Eukaryotic cell lines
☒	☐	Palaeontology and archaeology
☐	☒	Animals and other organisms
☒	☐	Clinical data
☒	☐	Dual use research of concern
☒	☐	Plants

Methods

n/a	Involved in the study	
☒	☐	ChIP-seq
☒	☐	Flow cytometry
☒	☐	MRI-based neuroimaging

Antibodies

Antibodies used

anti-MCY: YEASEN,catalog number 30601ES60, lot number M16071.anti-GAPDH : proteintech, catalog number 60004-1-Ig, lot number10020246.
Goat anti-mouse IgG(H+L)HRP: Sungene Biotech, China ,catalog number LK2003L, lot number AH261.

Validation

anti-MCY: https://www.yeasen.com/products/detail/108
anti-GAPDH :https://www.ptgcn.com/products/GAPDH-Antibody-60004-1-Ig.htm
Goat anti-mouse IgG(H+L)HRP: http://www.sungenebiotech.com/index.php?m=Product&a=product_xq_t&catid=2&proid=56&prid=79&pid=762&id=1648

Eukaryotic cell lines

Policy information about cell lines and Sex and Gender in Research

Cell line source(s)

HEK293 and HEK293T cells were purchased from Shanghai Institutes for Biological Sciences.

Authentication

HEK293 and HEK293T cell lines have been authenticated by morphology.

Mycoplasma contamination

Cell lines have no mycoplasma contamination.

Commonly misidentified lines
(See ICLAC register)

No commonly misidentified cell lines were used.

Animals and other research organisms

Policy information about studies involving animals; ARRIVE guidelines recommended for reporting animal research, and Sex and Gender in Research

Laboratory animals

Hartley guinea pigs (6-7 weeks) were purchased from the Nanjing Laifu (China). C57BL/6 mice (6-8 weeks) were purchased from Jiangsu Huachuang Sino (China). SD rats (8 weeks) were purchased from Jiangsu Huachuang Sino (China). All animals were maintained in a controlled environment (23 ± 2°C, 50 ± 10% humidity, 12 h light/dark cycle) with access to standard food and water.

Wild animals

Our study did not involve wild animals.

Reporting on sex

Consistent with previous findings, only males were used in the experiment.

Field-collected samples

The study did not involve sample collected from the field.

Ethics oversight

All procedures were carried out in accordance with the Guide for the Care and Use of Laboratory Animals and were approved by the ethics committee of the China Pharmaceutical University 2021-10-008 .

Note that full information on the approval of the study protocol must also be provided in the manuscript.
